# Pancreatic enzyme replacement therapy in advanced adenocarcinoma of the pancreas improved overall survival: a retrospective, single institution study

**DOI:** 10.1093/oncolo/oyaf014

**Published:** 2025-04-15

**Authors:** Vincent J Picozzi, Margaret T Mandelson, Anas Najjar, Moming Li, Diala E Harb, Jens J Kort

**Affiliations:** Virginia Mason Hospital and Medical Center, Seattle, WA 98111, United States; Benaroya Research Institute, Seattle, WA 98101, United States; Benaroya Research Institute, Seattle, WA 98101, United States; Data and Statistical Sciences, AbbVie Inc., North Chicago, IL 60064, United States; US Medical Affairs, AbbVie Inc., Mettawa, IL 60045, United States; US Medical Affairs, AbbVie Inc., Mettawa, IL 60045, United States

**Keywords:** advanced pancreatic ductal adeno Ca, PERT, exocrine pancreatic insufficiency, pancreatic cancer associated weight loss

## Abstract

**Background:**

Weight loss and exocrine pancreatic insufficiency are common in advanced pancreatic ductal adenocarcinoma (PDAC) and are associated with adverse outcomes. However, there is limited evidence on the impact of pancreatic enzyme replacement therapy (PERT) in patients with advanced PDAC.

**Patients and methods:**

We retrospectively studied 501 patients with advanced PDAC and exocrine pancreatic insufficiency from the Virginia Mason Pancreas Cancer Program Data Resource treated between 2010 and 2019 with first-line chemotherapy. Clinical outcomes were compared between those who received PERT and those who did not at 8 weeks after chemotherapy start.

**Results:**

In total 188 (38%) patients received PERT; 313 patients (62%) did not. PERT patients experienced less weight loss (–1.5 vs –2.5 kg, *P* = .04), less decline in the prognostic nutrition index −1.9 vs −3.0, *P* = .01), and a greater reduction in the additive score of the Patient-Generated Subjective Global Assessment (–8.4 vs –-6.0, *P* = .02). Importantly, median (95% CI) overall survival (OS) was significantly longer in the PERT vs non-PERT group (17.1 months vs 12.5 months, respectively *P* = .001), and the adjusted hazards ratio indicated superior median OS in patients prescribed PERT (HR = 0.73, *P* < .001).

**Conclusions:**

Our findings suggest that treatment of exocrine pancreatic insufficiency (EPI) in advanced PDAC is associated with improvements in nutrition and overall survival.

**Implications for practice**

Pancreatic cancer-associated weight loss is highly prevalent, and its pathophysiology is not well understood. A multi-disciplinary team approach is needed to address anorexia, malabsorption, malnutrition, and sarcopenia that will optimize patient outcomes. In this study, we provide further evidence for the importance of treatment of exocrine pancreatic insufficiency with adequate dosing of pancreatic enzyme replacement therapy (PERT). Adequate PERT corrects malabsorption and maldigestion with a meaningful impact on these patients’ lives.

## Introduction

In 2022, cancer of the pancreas was diagnosed in more than 62 000 individuals in the United States^1^ and 510 000 worldwide.^2^ It is currently the third leading cause of cancer death in the United States and is projected to be the second leading cause of cancer death by 2030.^3^ Five-year relative survival is approximately 12.5%.^4^ Most patients are diagnosed with pancreatic ductal adenocarcinoma (PDAC) at an advanced stage. Definitive surgical resection is generally a *sine qua non* for curative therapy, patients with inoperable or metastatic pancreatic cancer have a more dismal prognosis with median survival frequently measured in months.^5^ As such, treatment for advanced PDAC is appropriately focused on both quality and quantity of life.

Drug therapy, and in particular chemotherapy, is the mainstay of therapy for advanced PDAC given its systemic nature. Seminal clinical trials in metastatic PDAC using a variety of chemotherapy regimens (eg, gemcitabine/nab-paclitaxel,^6^ FOLFIRINOX,^7^ nal-IRIFOX^8^) have typically yielded median overall survival of 9-12 months. Large trials in locally advanced, generally unresectable PDAC report a median overall survival of 15-18 months.^9,10^ However, chemotherapy for these patients is often challenging in addition to being imperfect: 10%-20% of patients are unable to complete the first several months of therapy due to disease progression and/or therapeutic intolerance. Hematologic, neurologic, and gastrointestinal toxicities are frequent and significant challenges to optimum therapy.

Weight loss is common in pancreatic cancer. Many patients report weight loss prior to diagnosis and in advanced PDAC, weight loss frequently accelerates throughout the disease trajectory^11^ with progressive weight loss and loss of muscle mass during treatment linked to impaired functional status, decreased therapeutic tolerance, reduced response to therapy, increased risk of infection, and shorter overall survival.^12-14^ The pathophysiology of weight loss in advanced PDAC is often complex, involving diminished nutritional intake, suboptimal absorption of nutrients, and hypercatabolism due to cancer activity. The term PAWL (**P**ancreas Cancer-**A**ssociated **W**eight **L**oss) has been coined to refer to this problem.^15^

Exocrine pancreatic insufficiency (EPI), reported in 50%-90% of patients with advanced PDAC,^16^ is associated with weight loss.^17^ Pancreatic enzyme replacement therapy (PERT) in patients with PDAC and EPI is a long-standing recommendation according to the National Comprehensive Cancer Network (NCCN) guidelines.^18^ However, the impact of PERT on clinical outcomes in advanced PDAC is unclear. The aim of this study was to investigate the relation between PERT and weight change, nutrition status, and overall survival in a large cohort of patients with advanced PDAC treated at a single institution.

## Patients and methods

### Study overview and population

Patients with advanced PDAC (= unresectable PDAC) were identified through the Virginia Mason Pancreas Cancer Program Data Resource (VM-PCAP), an ongoing effort that integrates information across multiple platforms (medical records, laboratory data, radiology records, and pharmacy records) into a single unified record with detailed patient, tumor, treatment, and outcome data. Patients were eligible for the study if: (1) initial diagnosis of advanced PDAC was between January 1, 2010 and December 31 2019; (2) they had EPI based on a clinical diagnosis with documented evidence (ie, signs, and symptoms) by a treating physician, and/or abnormal fecal elastace-1 test (FE-1) (FE-1 ≤200 μg/g stool), and/or abnormal direct pancreatic function test; and (3) received all first-line chemotherapy treatment (Supplementary Table S1) up to initial staging event (at approximately 8 weeks) or longer at this institution. Patients under treatment for a concurrent cancer, with any initial treatment at an outside facility, or with unscheduled or delayed restage imaging, defined as less than 4 weeks or greater than 12 weeks from first treatment, were excluded from analysis.

PERT usage, ascertained by prescription date, was defined as use for at least 50% of interval from initial cancer treatment to first restaging at recommended dosing per package insert (500–2500 lipase units/kg/meal for a minimum of 3 meals/day). Patients were classified as no PERT use if there was no evidence of PERT prescription or PERT was prescribed following initial restaging event. Patients prescribed PERT at lower than recommended dose and/or use for <50% interval between first treatment and reassessment were excluded from study. PERT treatment of EPI was routinely more implemented during the later 5 years of the study (Supplementary Table S2).

### Endpoints and assessments

The primary endpoint was change in body weight from first treatment to initial cancer restage, at approximately 8 weeks of treatment. Changes in two measures of nutrition status during the same timeframe were investigated: (1) the Prognostic Nutritional Index,^19^ calculated from serum albumin levels and peripheral lymphocyte count and available on all study patients; and (2) change in additive score (box 1-4) from the Patient-Generated Subjective Global Assessment (PG-SGA) Short Form,^20^ a well-accepted patient-reported instrument for assessment of the nutritional status in cancer patients available on a subset of study patients for the period of 2014-2019. Overall survival was measured from the date of first treatment to the date of death or the last follow-up, whichever occurred first.

### Statistical analysis

For continuous variables, means ± SD or medians with range are reported. Continuous baseline characteristics were compared between subgroups using the independent samples *t*-test or Mann-Whitney *U*-test depending on their distribution. Categorical variables were compared using the chi-square test. For weight, PNI, and PG-SGA, analysis of covariance (ANCOVA) was used to compute change from baseline. For overall survival, the Kaplan-Meier method was used to compute survival probabilities and the Cox proportional hazards model was used for adjusted survival analysis. Age, race, sex, ECOG, PNI, NLR, and chemotherapy factors were used in all adjusted analyses. All *P*-values were 2-sided, with *P* < .05 considered statistically significant. Analysis was performed in R (V4.3.2, October 31, 2023).^21^

## Results

### Patient Characteristics

**Figure 1.**
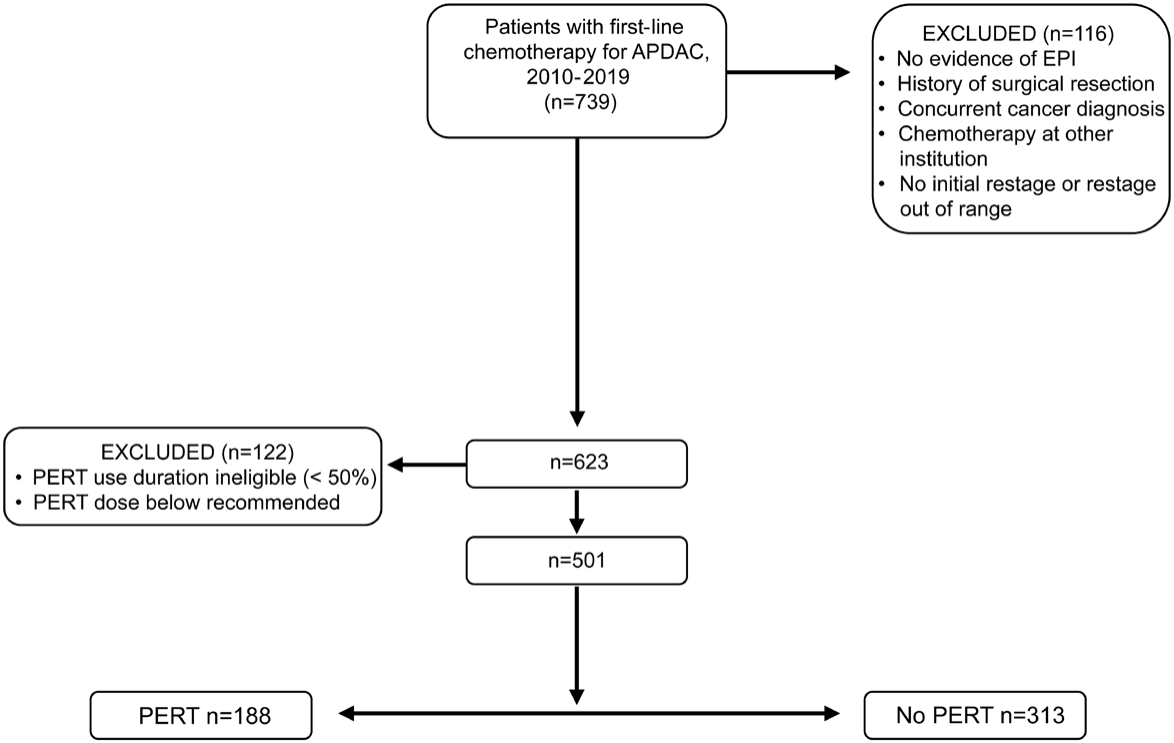
Patient disposition. Abbreviations: APDAC, advanced pancreatic ductal adenocarcinoma; EPI, exocrine pancreatic insufficiency; PERT, pancreatic enzyme replacement therapy.

Of the 739 patients with advanced PDAC initially treated at our institution between 2010 and 2019, 501 patients were eligible and included in the present study: 188 with PERT and 313 without PERT (Figure 1).

Patient characteristics at the start of treatment are shown in Table 1. Those receiving PERT were less favorable with respect to both frequency of weight loss prior to diagnosis (79.3% vs 64.9%, *P* = .001) and magnitude of weight loss (−9.8% vs −7.1 %, *P* < .001). Patients prescribed PERT were more favorable with respect to ECOG performance status (ECOG PS 0/1 96.8% vs 91.1%, *P* = .013). In addition to treatment, patient characteristics such as age, presence of diabetes, serum albumin, BMI class, and neutrophil/lymphocyte ratio (NLR) were not different between the 2 groups. In the PERT group the median prescribed PERT dose was 754 LU/kg/meal, or expressed as a median dose of 48 000 LU/meal.

**Table 1.** Baseline characteristics.

	PERT (*n* = 188)	No PERT (*n* = 313)	*P* value
Age, years, mean (SD)	67.0 (10.3)	67.3 (9.6)	.704
Sex, *n* (%)			
Male	98 (52.1)	167 (53.4)	.790
Female	90 (47.9)	146 (46.7)	
Race, *n* (%)			
White	178 (94.7)	276 (88.2)	.081
Black	2 (1.1)	7 (2.2)	
Asian	6 (3.2)	27 (8.6)	
Other/unknown	2 (1.1)	3 (1.0)	
ECOG performance status, *n* (%)			
0/1	182 (96.8)	285 (91.1)	.013
2/3	6 (3.2)	28 (9.9)	
BMI class, *n* (%)			
Underweight (<18.5)	4 (2.1)	10 (3.2)	.382
Normal (18.5-24.9)	87 (46.3)	134 (42.8)	
Overweight (25.0-29.9)	64 (34.0)	96 (30.7)	
Obese (≥30)	33 (17.6)	73 (23.3)	
Weight loss prior to diagnosis, *n* (%)	149 (79.3)	203 (64.9)	.001
Percent change in weight prior to diagnosis, mean (SD)	−9.8 (8.5)	−7.1 (7.7)	<.001
PG-SGA additive score [box 1-4], mean (SD)^*^	10.7 (6.1)	8.6 (7.4)	.01
Diabetes, *n* (%)	70 (37.2)	112 (35.8)	.744
PNI, mean (SD)	44.8 (5.4)	44.3 (4.8)	.39
NLR, mean (SD)	4.0 (2.6)	4.3 (3.2)	.239
Serum albumin g/dL, mean (SD)	3.7 (0.4)	3.7 (0.4)	.772
5FU-based therapy, *n* (%)	48 (25.7)	78 (24.9)	.852
Primary tumor location			
Head	123 (65.4)	169 (54.0)	
Body	27 (14.4)	58 (18.5)	
Tail	6 (3.2)	39 (12.5)	
Other (neck)	9 (4.8)	11(3.5)	
Overlapping lesion	21 (11.2)	36 (11.5)	
NOS	2 (1.1)	0	

^*^Available on 197 study patients: 104 PERT, 93 No PERT.

Abbreviations: 5-FU, 5-Fluorouracil; BMI, body mass index; ECOG, Eastern Cooperative Oncology Group; NLR, Neutrophil to lymphocyte ratio; PNI, prognostic nutritional index; SD, standard deviation

### Nutritional and clinical outcomes

**Figure 2.**
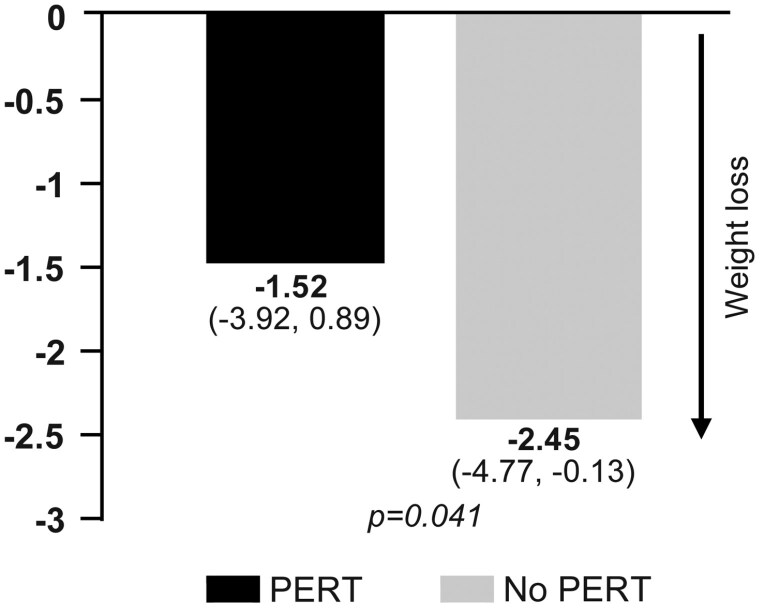
Adjusted change in mean (95% CI) body weight (kg) from initial treatment to first restage in PERT and non-PERT groups.

**Figure 3.**
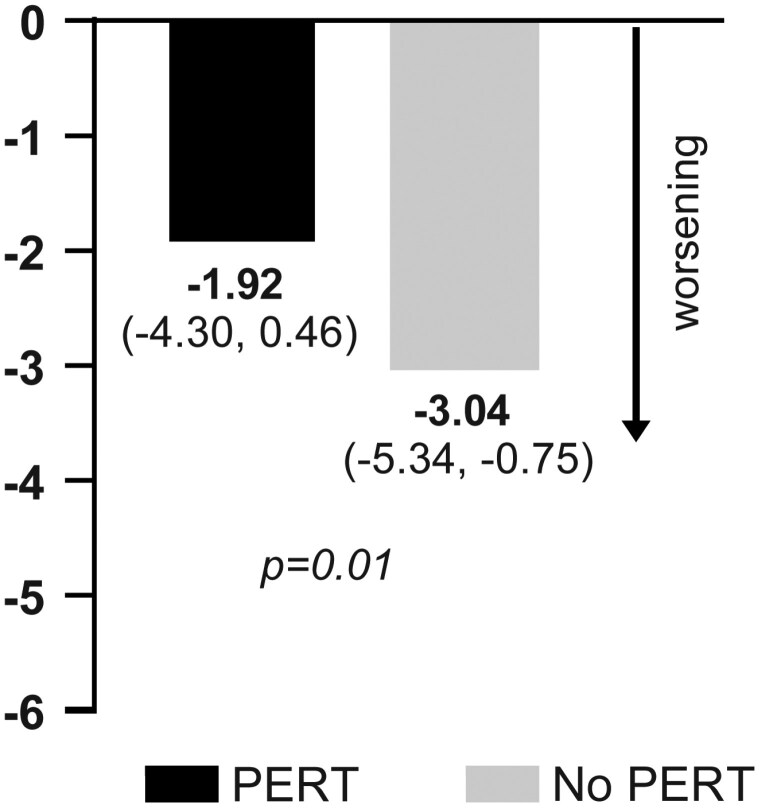
Adjusted change in mean (95% CI) prognostic nutritional index (PNI) from initial treatment to first restage in PERT and non-PERT groups.

Patients prescribed PERT experienced less weight loss at approximately 8 weeks following diagnosis in both unadjusted (not shown) and adjusted analysis (Figure 2). Additional adjustments for the year of diagnosis did not alter these findings. The favorable effect of PERT in patients with advanced PDAC was also observed for change in PNI in both unadjusted (not shown) and adjusted analysis (Figure 3).

**Figure 4.**
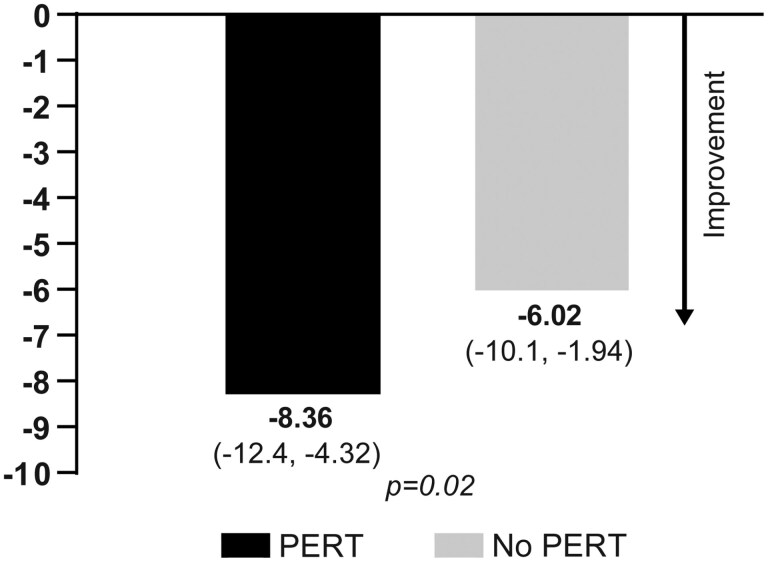
Adjusted change in mean (95% CI) PG-SGA additive score from initial treatment to first restage in PERT and non-PERT groups.

One hundred ninety-seven patients underwent paired PG-SGA assessments during the approximate 8-week period being studied. The change from baseline to week 8 in additive PG-SGA (−8.4 vs −6.0, *P* = .02 -lower score is better) was statistically superior in the PERT vs non-PERT groups in both unadjusted analysis (not shown) and adjusted analysis (Figure 4). When considering each of the 4 elements that contribute to the overall PG-SGA score (weight, food intake, symptoms preventing eating, and activities preventing eating), the basis for favorable change largely was derived from symptoms preventing eating (−3.83 vs −2.40, *P* = .03). The other 3 elements, while showing improvement from baseline to 1st reassessment, failed to achieve statistical significance. PERT also had a favorable effect on therapy administration and therapeutic outcomes.

Importantly, the impact of PERT was additionally seen in therapeutic outcomes Table 2). With respect to therapy administration, 84% of PERT patients vs 76% of non-PERT patients (*P* = .05) received intended chemotherapy treatments during the first 8 weeks of therapy. This may have been due to improved gastrointestinal symptomatology observed.

**Table 2.** Therapeutic outcomes^*^ at initial restaging.

	PERT (*n* = 188)	No PERT (*n* = 313)	*P-*value
Received intended cycles of therapy, *n* (%)	155 (82.4)	236 (76.0)	.08
CTC grades 3 and 4 toxicity, *n* (%)	14 (7.5)	29 (9.3)	.60
Toxicity-related hospitalization, *n* (%)	11 (5.9)	13 (4.2)	.28
Percent change in CA 19-9, mean (SD)	-54.4 (56.9)	7.0 (453.1)	.14
Radiographic response partial/stable, *n* (%)	161 (85.6)	245 (78.3)	.09

^*^Adjusted for age, race, sex, ECOG, NLR, PNI, and chemotherapy.

**Figure 5.**
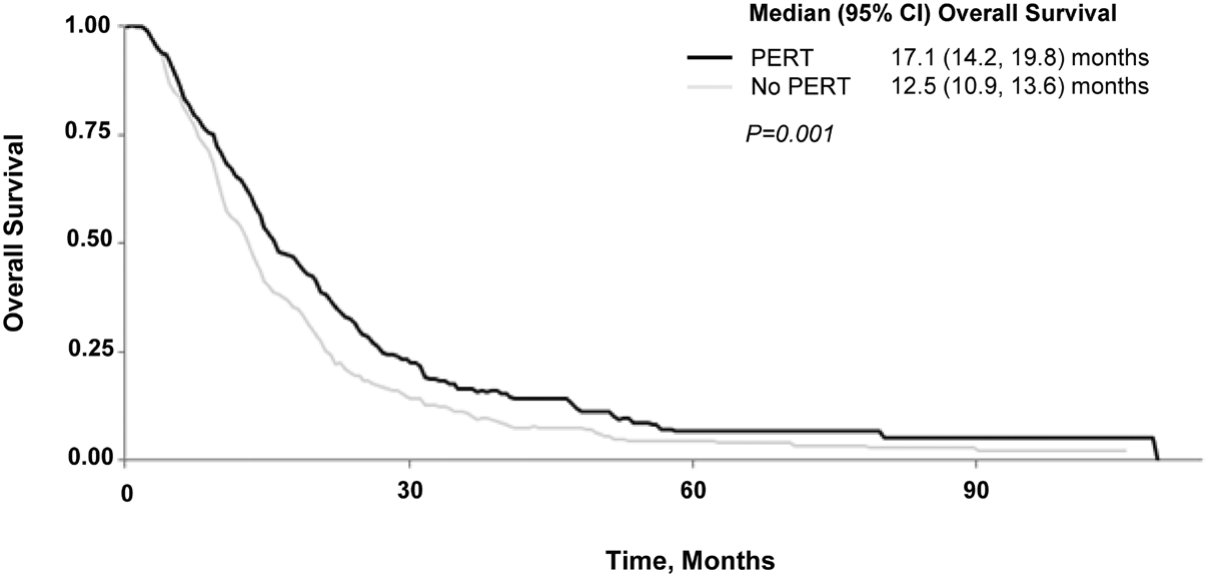
Unadjusted median overall survival in PERT vs no-PERT groups.

PERT was also associated with a survival benefit. Median (95% CI) overall survival was significantly longer in the PERT vs non-PERT group (17.1 months (95% CI 14.2-19.8 months) vs 12.5 months 95% CI 10.9-13.6 months, *P* = .001, Figure 5). An adjusted analysis by Cox regression provided a hazard ratio for PERT use of 0.73 (95% CI: 0.60-0.88, *P* = .001). Survival probability (95% CI) at 6, 12, and 24 months for PERT vs. non-PERT patients was 0.85 (80%-90%) vs 84% (80%-88%), *P* = .67, .69 (63%-76%) vs .51 (46%-57%), *P* < .001 and .34 (27%-41%) vs .19 (15%-24%, *P* < .001, respectively (Supplementary Table S3). Non-significant trends were observed in additional adjusted analysis suggesting that PERT patients may be more likely to complete intended cycles of therapy (82% vs 76%, *P* = .08) and have a stable or partial radiographic response (86% vs 78%, *P* = .09). No difference in frequency of grades 3 and 4 toxicities by Common Terminology Criteria for Adverse Events^22^ or toxicity-related hospitalizations was observed (Table 2).

## Discussion

Weight loss is endemic to pancreatic cancer and is a matter of immense importance to both patients and caregivers. The approach to this problem requires multidimensional thought. One useful construct is to consider this problem according to 3 major components: (1) decreased caloric intake/anorexia associated with a host of gastrointestinal and other symptoms, (2) caloric malabsorption/EPI, and (3) cancer cachexia/sarcopenia. The final component involves consideration of the cancer treatment itself, mitigation of its metabolic and inflammatory consequences, and optimum physical activity and exercise. Along with PDAC itself, both treatment and non-treatment-related morbidities often influence this problem. It is also noteworthy that these factors affect a wide range of organ systems producing immunological, endocrinological, gastrointestinal, musculoskeletal, and neuropsychiatric pathophysiology. As such, patients with advanced PDAC typically require a complicated and largely individualized approach to weight loss.

Given the high prevalence of EPI in PDAC, the use of PERT is generally recommended in major pancreatic cancer guidelines^18,23^ However, clinical experience in support of this position, largely based on retrospective non-randomized data, has produced variable results, especially in advanced PDAC. Dominguez-Munoz et al.^24^ performed a retrospective, nonrandomized study of 160 unresectable PDAC patients. Seventy-four underwent additional analysis of pancreatic function; 49/74 (66%) received PERT as a result. Overall survival for this group was superior to the 86 patients who did not (189 days vs 95 days, HR 2.12, *P* < .001). Saito et al^25^ performed a prospective study of 91 advanced PDAC patients receiving chemotherapy. The incidence of a positive N-benzoyl-tyrosyl para-aminobenzoic acid test at diagnosis was found to be high (94%). The use of PERT was found to have a favorable effect on BMI (*P* < .001) and median overall survival in univariate analysis, although this benefit disappeared in multivariate analysis. Zdenkowski et al^26^ performed a randomized study of PERT usage in advanced PDAC patients receiving chemotherapy, however, no meaningful results could be achieved due to poor enrollment. Finally, Giordano et al^27^ compared PERT along with a variety of dietary interventions (including feeding support) in 106 APDAC patients receiving abraxane-containing first-line chemotherapy. The experimental group experienced a much higher median overall survival (16.5 vs 7.5 months, *P* < .001). However, this study was hindered by small samples size, a much higher percentage of the experimental group receiving a 4 drug (PAXG) as opposed to a 2 drug (GA) treatment regimen, and the use of supplemental nutrition in a nonstandard way.

A recent report by Klassen et al^28^ also looked at the question of PERT use in advanced PDAC, in particular with respect to the question of PERT dose. In this retrospective study, 210 patients with APDAC were divided into 3 groups: (1) no PERT use (129 patients), (2) low dose PERT (40 patients < 75 000 USP lipase units) and high dose PERT (41 patients, > 75 000 lipase units/day). There were no significant differences in baseline characteristics between high-dose and low-dose groups. Muscle loss was more prevalent among low dose compared to both high-dose and No PERT groups (88% vs 58% and 67%, *P* < .05). In the multivariable model predicting muscle loss, low-dose PERT was independently associated with greater odds of muscle loss (OR 5.4, *P* = .004) vs high dose, independent of tumor response, disease stage, and chemotherapy regimen. However, in this study, there was no significant difference in muscle mass loss between a no-PERT group and high-dose PERT-group, and no information was provided on the EPI status of patients.

The study reported here adds to the existing literature and provides added support for the use of PERT in patients with advanced PDAC and EPI. Notably, although still retrospective, this is a much larger study, containing more patients than the above studies combined, with more detailed observations. Although the patient groups were somewhat imbalanced with respect to stage and performance status, the overall nutritional deficit of the PERT patients was greater than the non-PERT patients at presentation. PERT seemed to produce favorable effects on patient nutritional status as expressed in changes in PNI, and with that, PERT use was seen to decrease the amount of weight loss in these patients. Moreover, PERT brought about a significant improvement in eating-related symptoms according to the paired PG-SGA data Box 3, and a trend for the ability to receive the planned chemotherapy during the first 8 weeks of treatment. Chemotherapy-related toxicity avoidance and clinical response were at least as good in PERT versus non-PERT patients. Perhaps most importantly, a significant difference in median overall survival was seen in PERT versus non-PERT patients, one that rivals the improvement in overall survival seen in many of the important chemotherapy trials cited above, and the difference in overall survival probability increased with time (Supplementary Table S3), and the PERT benefit remained significant following propensity-score matching of PERT vs no PERT patients (Supplementary Figure S1).

This study has inherent limitations given its retrospective, single-institution character. Imbalances were seen in patient characteristics (percentage weight loss, stage, and performance status), and PERT use was more common during the later years of recruitment (2015-2019). Importantly, the survival benefit in PERT vs no PERT patients remained significant in analyses adjusted for year of enrollment as covariate (data not shown). Issues exist with respect to PERT dosing; the prescribed dose of PERT was used here and actual PERT use by the patients is not known, and it is similarly unclear whether the median prescribed PERT dose of 754 LU/kg/meal or expressed as a median dose of 48 000 LU/meal is optimal. Concomitant nutritional intake was not analyzed. An ideal study would be a prospective multi-institutional randomization with and without PERT mindful of the above limitations. However, such a study might well be considered unethical given the current data, thus utilization of imperfect data is required.

The biggest limitation to this, or perhaps any study of malnutrition in advanced PDAC, is the inability of any single intervention to display optimum effect unless all of the other major factors influencing malabsorption in that particular patient are addressed. To this end, a variety of investigational approaches to weight loss and cachexia are being explored in advanced PDAC that might leverage the beneficial effects of PERT.

Reduced caloric intake is common; over 50% of patients have moderately or severely reduced food intake at diagnosis, leading to severe weight loss and reduced survival. With respect to appetite stimulation, the use of megestrol acetate or multiple investigational agents such as ghrelin agonists (amamorelin approved in Japan)^29^ GDF15-GFRAL inhibitors,^30^ and melanocortin 4 receptor antagonists.^31^

The recently reported study by Groarke et al^29^ deserves particular note. In this 187-patient, randomized, double-blinded, phase II trial, 45 patients with PDAC cachexia as defined by Fearon et al^32^ and GDF-15 levels greater than 1500 pg/ml received ponsegromab (an anti-GDF-15 antibody). The drug was given subcutaneously once every 4 weeks at doses up to 400 mg for 12 weeks. The use of PERT was not protocol-specified. Patients experienced increasing weight gain proportional to ponsegromab dose. In 10 patients with PDAC receiving the 400 mg dose, weight gain at 12 weeks was 2.52 kg (95% PI −0.12 to 4.95 kg) better than control. Improvements in appetite, cachexia symptoms, and activity level were also seen. If these results are confirmed by additional observations, ponsegromab potentially represents a major advance in reversing cancer cachexia in PDAC.

In addition to overall improvement in protein-caloric intake, specific support of nutrient intake that is rate-limiting in maintaining key metabolic pathways may be of particular benefit. One example is omega-3 fatty acids which have been proposed as anti-inflammatory mediators, thereby reducing the activation of proteolytic pathways and increasing appetite.^33^ Also, supplementation of branched-chain amino acids such as arginine (often deplete in cancer cachexia) may favorably modulate the immune system while preserving muscle mass and improving overall nutrition and survival.^34^

Regarding cancer cachexia and sarcopenia, a complex system of signaling pathways coordinates muscle protein balance including an anabolic arm reliant on growth factors and nutrient signaling along with contractile activity. A variety of cytokine mediators, most particularly IL-6, but also Il-1, TNF, INF-ɤ, and others promote muscle wasting.^35^ Also, chemotherapy itself is also associated with muscle loss. For example, a recent study in pancreatic cancer showed an 11% mean decrease in muscle mass over 3 months in patients receiving FOLFIRINOX,^36,37^ an amount of muscle loss comparable to 20-25 years of aging.

Given the above, multimodal strategies as practical in conjunction with PERT would seem required to combat weight loss and cachexia in advanced PDAC. A study in Japan demonstrated the feasibility of nutritional counseling, psychosocial support, and exercise training as part of first-line therapy of advanced PDAC.^38^Additionally, the MENAC trial (NCT02330926) trial was recently reported.^39^ In this trial, 212 patients with stage III lung or stage IV pancreatic cancer were randomized to standard treatment for their cancer versus standard treatment plus dietary guidance, ONS-containing EPA (oral nutritional supplementation using an eicosapentanoic acid), daily ibruprofen, and a home-based exercise program. Over 6 weeks, weight stabilized in patients assigned to multimodal treatment compared with those assigned to standard care (mean weight change [SD] 0.05 kg [3.8] vs –0.99 kg [3.2], respectively) with a mean difference in weight change of −1.04, 95% CI −2.02 to −0.06, *P* = .04. There was no conclusive difference in muscle mass (mean change [SD] −6.5 cm^2^ [10.1] vs −6.3 cm^2^ [11.9], *P* = 0.93) or in mean step counts [SD] (−377.7 [2075] vs −458 [1858], *P* = 0.89). Furthermore, the method of PERT usage was not specified in this trial.

## Conclusions

These data, with the limitations of a retrospective cohort study, support the treatment of EPI with PERT in advanced PDAC given its potential benefits not just to nutritional status, but also to treatment tolerance, quality of life, and overall survival. Extension of these observations seems appropriate, with particular attention to an improved mechanistic understanding of the impact of PERT in combination with other nutritional therapies.

## Supplementary Material

oyaf014_suppl_Supplementary_Tables_1

oyaf014_suppl_Supplementary_Tables_2

oyaf014_suppl_Supplementary_Tables_3

oyaf014_suppl_Supplementary_Figures_1

## Data Availability

The data underlying this article are available in the article and in its online supplementary material.
