# Supplementary material for: Pancreatic enzyme replacement therapy in advanced adenocarcinoma of the pancreas improved overall survival: a retrospective, single institution study
Source: Oncologist. 2025 Apr 15;30(4):oyaf014. doi: 10.1093/oncolo/oyaf014 (PMC11997656; doi:10.1093/oncolo/oyaf014)
Supplement: oyaf014_suppl_Supplementary_Tables_1 [file oyaf014_suppl_supplementary_tables_1.docx]

**Supplement to Pancreatic Enzyme Replacement Therapy in Advanced Adenocarcinoma of the Pancreas Improved Overall Survival: A Retrospective, Single Institution Study**

**Supplementary Table 1. First-course chemotherapy regimens in study sample**

| Regimen | % sample received  (N = 501) |
| --- | --- |
| Gemcitabine based combination | 92.6 |
| 5-FU based combination | 4.2% |
| Single agent Gemcitabine | 1.6% |
| Other | 2.6% |

5FU = 5-fluorouracil.
