# Supplementary material for: Pancreatic enzyme replacement therapy in advanced adenocarcinoma of the pancreas improved overall survival: a retrospective, single institution study
Source: Oncologist. 2025 Apr 15;30(4):oyaf014. doi: 10.1093/oncolo/oyaf014 (PMC11997656; doi:10.1093/oncolo/oyaf014)
Supplement: oyaf014_suppl_Supplementary_Tables_2 [file oyaf014_suppl_supplementary_tables_2.docx]

**Supplement to Pancreatic Enzyme Replacement Therapy in Advanced Adenocarcinoma of the Pancreas Improved Overall Survival: A Retrospective, Single Institution Study**

| **Supplementary Table 2. Patient distribution by 2010-2014 and 2015-2019** | | | | | | | |
| --- | --- | --- | --- | --- | --- | --- | --- |
|  | PERT | | | No-PERT | | All | |
| year_rx1 | | n | % | n | % | n | % |
| 2010-2014 | | 34 | 18.1 | 168 | 53.7 | 202 | 40.3 |
| 2015-1019 | | 154 | 81.9 | 145 | 46.3 | 299 | 59.7 |
| Total | | 188 | 100 | 313 | 100 | 501 | 100 |

Rx = treatment.
