# Supplementary material for: Pancreatic enzyme replacement therapy in advanced adenocarcinoma of the pancreas improved overall survival: a retrospective, single institution study
Source: Oncologist. 2025 Apr 15;30(4):oyaf014. doi: 10.1093/oncolo/oyaf014 (PMC11997656; doi:10.1093/oncolo/oyaf014)
Supplement: oyaf014_suppl_Supplementary_Tables_3 [file oyaf014_suppl_supplementary_tables_3.docx]

**Supplement to Pancreatic Enzyme Replacement Therapy in Advanced Adenocarcinoma of the Pancreas Improved Overall Survival: A Retrospective, Single Institution Study**

| **Supplementary Table 3. Unadjusted survival probability over time** | | | |
| --- | --- | --- | --- |
| Months after initiation of chemotherapy | No-PERT (n=313)  Survival Probability (95% CI) | PERT (n=188)  Survival Probability (95% CI) | *P -* value |
| 6 | 0.84 (0.8 – 0.88) | 0.85 (0.80 – 0.90) | .667 |
| 12 | 0.51 (0.46 – 0.57) | 0.69 (0.63 – 0.76) | <.001 |
| 18 | 0.34 (0.29 – 0.39) | 0.47 (0.41 – 0.55) | .003 |
| 24 | 0.19 (0.15 – 0.24) | 0.34 (0.27 – 0.41) | .001 |
| 30 | 0.14 (0.10 – 0.18) | 0.25 (0.19 – 0.32) | .003 |
| 36 | 0.11 (0.08 – 0.15) | 0.17 (0.12 – 0.23) | .064 |
| CI = confidence interval; PERT = pancreatic enzyme replacement therapy. | | | |
