# Supplementary material for: Pancreatic enzyme replacement therapy in advanced adenocarcinoma of the pancreas improved overall survival: a retrospective, single institution study
Source: Oncologist. 2025 Apr 15;30(4):oyaf014. doi: 10.1093/oncolo/oyaf014 (PMC11997656; doi:10.1093/oncolo/oyaf014)
Supplement: oyaf014_suppl_Supplementary_Figures_1 [file oyaf014_suppl_supplementary_figures_1.docx]

**Supplementary Figure 1. Kaplan-Meier survival probability estimates based on a propensity score matched cohort**


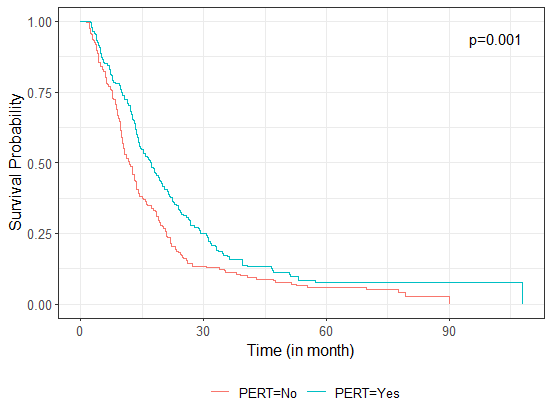


|  | n | events | median | 95% LCL | 95% UCL |
| --- | --- | --- | --- | --- | --- |
| PERT = No | 188 | 179 | 11.8 | 10.3 | 13.6 |
| PERT = Yes | 188 | 165 | 17.1 | 14.2 | 19.8 |

Subjects were matched for baseline age, sex, race, weight, ECOG status, NLR, PNI, and chemotherapy. The Kaplan-Meier method was used to compute survival probabilities and a Cox proportional hazards model with PERT as the only covariate was used for the analyses. A hazard ratio of 0.7 for the PERT effect with 95% CI (0.57, 0.87) was obtained based on this propensity score matched cohort.
